# Ultrafine Jujube Powder Enhances the Infiltration of Immune Cells during Anti-PD-L1 Treatment against Murine Colon Adenocarcinoma

**DOI:** 10.3390/cancers13163987

**Published:** 2021-08-07

**Authors:** Nan Jing, Luoyang Wang, Huiren Zhuang, Guoqiang Jiang, Zheng Liu

**Affiliations:** 1 Key Lab of Industrial Biocatalysis Ministry of Education, Tsinghua University, Beijing 100084, China; jn17@mails.tsinghua.edu.cn (N.J.); zhuanghr19@mails.tsinghua.edu.cn (H.Z.); 2 Department of Chemical Engineering, Tsinghua University, Beijing 100084, China; 3 School of Basic Medicine, Qingdao University, Qingdao 266071, China; wangnan235@qdu.edu.cn

**Keywords:** immune checkpoint inhibitors, colon adenocarcinoma, dietary intervention, short-chain fatty acids (SCFAs), tumor-infiltrating lymphocytes

## Abstract

**Simple Summary:**

While modulating gut microbiota using dietary intervention with natural nutrients has proven to be effective in improving the response rate of immune checkpoint inhibitors (ICIs), the underpinning mechanism is poorly understood. This work demonstrates that the oral administration of ultrafine jujube powder (JP) let to a significant alteration of gut microbiota, an increased abundance of Clostridiales, including Ruminococcaceae and Lachnospiraceae, an elevated SCFA production, an intensified infiltration of CD8^+^ T cells to the tumor microenvironment, and a greatly improved response of anti-PD-L1 treatment against murine colon adenocarcinoma. Moreover, the size of the JP particles had a significant impact on the abovementioned attributes. The present study demonstrates that dietary intervention with nutrients is highly effective in modulating the gut microbiota for an improved immune checkpoint blockage therapy.

**Abstract:**

Whereas dietary intervention with natural nutrients plays an important role in activating the immune response and holds unprecedented application potential, the underpinning mechanism is poorly understood. The present work was dedicated to comprehensively examine the effects of ultrafine jujube powder (JP) on the gut microbiota and, consequentially, the effects associated with the response rate to anti-PD-L1 treatment against murine colon adenocarcinoma. A murine colon adenocarcinoma model with anti-PD-L1 immunotherapy was established to evaluate how dietary interventions affect the microbiota. In vitro and in vivo experiments confirmed the role of SCFAs in the immune response. Oral administration of JP greatly improves the response of anti-PD-L1 treatment against murine colon adenocarcinoma. Such an improvement is associated with the alteration of gut microbiota which leads to an increased abundance of Clostridiales, including Ruminococcaceae and Lachnospiraceae, an elevated SCFA production, and an intensified infiltration of CD8^+^ T cells to the tumor microenvironment. This work demonstrates that JP is particularly effective in modulating the gut microbiota for an improved immune checkpoint blockage therapy by boosting cytotoxic CD8^+^ T cells in tumor-infiltrating lymphocytes. The experimental findings of the present study are helpful for the development of dietary intervention methods for cancer immunotherapy using natural nutrients.

## 1. Introduction

While immune checkpoint inhibitors (ICIs) targeting programmed cell death ligand 1 (PD-1) or cytotoxic T lymphocyte-associated protein-4 (CTLA-4) have achieved tumor regression in several cancers [1,2], the unexpected low response rate, typically below 30%, hinders the full display of this revolutionary procedure [3]. Recent years have witnessed ever growing efforts in modulating the gut microbiome for improving responses to ICIs [4,5,6,7,8,9], through a wide variety of innovative strategies such as dietary interventions [10], administration of bacterial consortia or “Designer Probiotics” [11], and fecal microbiota transplantation (FMT) [12].

Among them, dietary intervention is practically attractive due to its public acceptability, natural availability, and quick effect [13,14]. Various microbial communities are intimately involved in human digestion and nutrient extraction [15]. It has been well established that the increased abundance of pectin, inulin, and alike is conductive to an enhanced response to ICI treatment [16], whereas the administration of natural ingredients rich in dietary fibers is effective to modulate the gut microbiota to a favorable pattern. Jujube (Chinese date), scientifically known as *Ziziphus jujuba* Mill., is a natural fruit extensively applied to traditional Chinese Medicine. Jujube delivers significant nutritional and medicinal values [17,18], such as antioxidant and anti-inflammatory effects [19], antimicrobial activity [20], anticancer properties [21], immunostimulating properties [22,23,24], and gastrointestinal protective activity [25]. In previous work, we demonstrated that the oral admission of jujube enriched the abundance of Lachnospiraceae while it reduced that of Prevotellaceae, thereby improving the therapeutic efficiency and response rate of anti-PD-L1 against murine colon adenocarcinoma [26].

The beneficial effects of natural nutrients may derive from the fermentation of dietary fibers into bioactive metabolites [27] that impact local and systemic immune responses [28], including the CD8^+^ T cell responses [29]. Jian and coworkers [30] confirmed that *L. murinus* is associated with the activation of intestinal DCs while *B. uniformis* displays the strongest positive correlation with the proportion of IFNγ^+^ CD8^+^ T cells in mesenteric lymph nodes (MLNs) in mice, which can trigger an antitumor immune response. Honda and coworkers [31] found that the abundance of *Bifidobacterium* is positively correlated with intratumoral and peripheral CD8^+^ T cell responses. Moreover, it has been shown that 11 specific bacterial strains from healthy donor stools could accumulate and recruit intestinal IFNγ^+^ CD8^+^ T cells [31]. McCoy and coworkers [32] confirmed that *Bifidobacterium pseudolongum* and *Lactobacillus johnsonii* could significantly enhance the CD8^+^ T cell responses in the tumor microenvironment (TME) consistent with the improved efficacy of anti-PD-L1 and anti-CTLA-4 therapy on colorectal cancer.

High concentrations of fecal or plasma SCFAs have been associated with better responses to PD-1 treatment and longer progression-free survival [33]. SCFAs play a vital role in communication between the gut microbiota and the mucosal immune system [34]. In animal studies, SCFAs have been shown to influence intestinal adaptive immune responses through regulating the size and function of the regulatory T-cell pool [35]. SCFAs can also amplify the cytotoxicity of CD8^+^ T cells through cellular metabolism, which further maintains a balance between innate and adaptive immunity [29].

This enhancement can be achieved by using natural nutrients (e.g., jujube powder) through nurturing the gut microbiota, simulating SCFAs, and enhancing infiltration of immune cells to the microenvironment of the tumor cell. Moreover, we conjecture that the infiltration efficiency can be enhanced by tuning both food composition and morphology. 

The Chinese classical medical books “The Theory of Food” (Shi Lun) by Hua Tuo and “Thousand Golden Preions” (Qian Jin Yao Fang) by Sun Simiao both recommended to “chew carefully and swallow slowly” so as to physically disrupt food as completely as possible. It is generally recognized that the most important impact of particle size on nutrition is the release and intestinal absorption of nutrients in the food. For instance, the hydration and other physicochemical properties of cellulose can be improved by reducing the size [36,37]. Meanwhile, growing evidence indicates that the nutritional value of food is not limited to the solubilized parts; it includes also those insolubilized components that can be used by gut microbes. Given the aforementioned findings, we hypothesize that the improved efficiency of anti-PD-L1 against murine colon adenocarcinoma attained with the aid of a favorable gut microbiota might be associated with an increased infiltration of immune cells in the TME. Moreover, we conjecture that the infiltration efficiency can be enhanced by tuning both the food composition and morphology. Here, we examined the effects of the size of JP particles on the metabolism of SCFAs, the composition of intestinal flora, and the infiltration of immune cells. We started with the investigation of the JP size effect on mice gut microbiota. We examined the JP particles in terms of the surface properties such as zeta potential, morphology, and aggregation. We applied JP in the anti-PD-L1 treatment against murine colon adenocarcinoma and determined the concentration of SCFAs, as well as the infiltration of CD8^+^ and CD8^+^INFγ^+^ in the microenvironment of tumor cells. These investigations allowed us to establish a comprehensive description of the underlying mechanism of JP in terms of the response rate to anti-PD-L1 treatment.

## 2. Materials and Methods

### 2.1. Materials

*Ziziphus jujuba* cv. Junzao was purchased from Akesu, Xinjiang, China. In vivo MAb anti-mouse PD-L1 (B7-H1) and immunoglobulin G (IgG, BE0090) were obtained from BioXCell (West Lebanon, NH, USA). All antibodies were bought from eBioscience (San Diego, CA, USA). Heat-resistant amylase, protease, and amyloglucosidase were purchased from Sigma-Aldrich (St. Louis, MO, USA). Reagents for cell culture were purchased from Gibco (Grand Island, NY, USA). Acetate, propionate, butyrate, and valerate were purchased from Macklin (Shanghai, China). All other chemicals and reagents were analytical grade, unless otherwise specified.

### 2.2. Preparation of Jujube Powders

*Ziziphus jujuba* cv. Junzao fruits were cleaned, sliced, and air-dried at 50 °C for 24 h, followed by coarse grinding using a pulverizer and sieving through a 100 mesh sieve, yielding jujube powder denoted as P. The ultrafine ground jujube powder was prepared at ultra-low temperature using an ultrafine grinder PLS-10L (Jinan Preshen Machinery Equipment Co., Ltd., Jinan, China), and then sieved through 200 mesh, 270 mesh, and 800 mesh sieves, yielding ultrafine powders denoted as S1, S2, and S3. All jujube powders were stored at −20 °C until analyzed.

### 2.3. Analysis of Particle Size and Surface Properties of Jujube Powders

The morphology of jujube powders was monitored using a JSM7401 scanning electron microscope (SEM) (JEOL, Tokyo, Japan). Zeta potential and particle size distribution were determined by dynamic light scattering (DLS) using a Mastersizer 3000 (Malvern instrument Ltd., Worcestershire, UK). Surface groups and chemical properties of jujube powders were determined with Fourier-transform infrared spectroscopy (FTIR) (Nicolet 6700FTIR, Thermo Scientific, Waltham, USA). The water content was determined with differential scanning calorimetry (DSC) (Q5000IR, TA Instruments, New Castle, PA, USA) in N_2_ atmosphere at a flow rate of 50 mL/min, whereas the temperature range was from 25 to 300 ℃ at a heating rate of 20 ℃/min, with an aluminum crucible as the reference. The rose Bengal staining method was used to detect the hydrophobicity of JP. Certain amounts (1–5 mg/mL) of JP and Bangladesh rose (RB, 10 μg/mL) were added to deionized water and stirred for 24 h (37 °C, 100 rpm). The concentration of supernatant was measured at 542.7 nm. The partitioning quotient (PQ) of RB was calculated according to the following formula: $\mathrm{PQ}=\frac{\mathrm{mass}\;\mathrm{of}\;\mathrm{RB}\;\mathrm{on}\;\mathrm{JP}\;\mathrm{surface}}{\mathrm{mass}\;\mathrm{of}\;\mathrm{RB}\;\mathrm{in}\;\mathrm{aqueous}\;\mathrm{phase}}\times 100\%$. Linear fitting was carried out with the content of JP as the abscissa and PQ as the ordinate. Therefore, the slope K was the adsorption rate of RB on the surface of JP, which increased with the magnitude of hydrophobicity.

### 2.4. Analysis of Dietary Fiber

The enzymatic–gravimetric method was used to determine the contents of total dietary fiber (TDF), soluble dietary fiber (SDF), and insoluble dietary fiber (IDF) in jujube powders according to the Association of Official Analytical Chemists (AOAC, 2002). Briefly, all samples were successively digested with amylase, protease, and amyloglucosidase to remove protein and starch. Specific experimental methods were based on AOAC with minor modifications [38].

### 2.5. Animals and Treatments

All mice used in experiments were 7 week old female C57BL/6 mice (Vital River Laboratory Animal Technology Co., Ltd., Beijing, China) housed under specific pathogen-free conditions according to the protocols approved by the Institutional Animal Care and Use Committees of Tsinghua University for animal welfare (approval ID SYXK2019-0037). 

The mouse model of colon cancer was established by subcutaneously inoculating mice with 5 × 10^5^ MC38 cells at the right flank after 1 week of adaptation. Tumor volumes (width^2^ × length)/2) were measured twice a week. Seven days after tumor inoculation, mice were divided into different groups of six mice with randomized tumor size. For the αPD-L1-treated groups (αPD-L1), mice received 200 μg of αPD-L1 by intraperitoneal injection twice on days 7 and 10. The isotype control group (CTR) mice received IgG at the same time. For groups treated with P, S1, S2, and S3, mice were gavaged with a dose of 800 mg/kg jujube powder daily from day 7 to day 25, following previous studies [39,40]. For the group treated with butyrate, butyrate at a dose of 80 mM was added to drinking water from day 7 to day 25. All mice were euthanized on day 25 for data analysis. MC38 cells were obtained from the American Type Culture Collection and were cultured in DMEM culture medium supplemented with 10% fetal bovine serum (FBS) and 1% penicillin–streptomycin at 37 °C in 5% CO_2_ atmosphere.

For short-chain fatty acid (SCFA) experiments, healthy mice were randomly divided into different groups of six mice after 1 week of adaptation. Acetate (C2), propionate (C3), and butyrate (C4) at a dose of 80 mM and mixtures of SCFAs (C2, C3, and C4 at a dose of 40 mM) were added to drinking water for 1 week. For microbial diversity and RNA-sequencing analysis, healthy mice were randomly divided into different groups of six mice after 1 week of adaptation and gavaged with P, S1, S2, or S3 at a dose of 800 mg/kg daily for 1 week. Eventually, all mice were euthanized on day 7 for data analysis. 

### 2.6. Lymphocyte Culture In Vitro and Proliferation Assay

The experimental methods were according to Cavaglieri with slight adjustment [41]. Briefly, mesenteric lymph nodes were isolated from healthy mice and prepared into a single-cell suspension with RPMI culture medium containing 10% FBS, 2 mM glutamine, and 1% penicillin–streptomycin. Lymphocytes (5 × 10^5^) were cultured in 96-well round-bottom culture plates. SCFAs (final concentration acetate (C2) 1 mM, propionate (C3) 1 mM, and butyrate (C4) 1 mM) and T cell mitogen Con A (final concentration 5 μg/mL) were added and then the final volume was adjusted to 200 μL for incubation (37 °C, 5% CO_2_). After 24 h or 48 h, the cells were then harvested for analysis of CD8^+^ and CD4^+^ T cells by FACS.

### 2.7. Analysis of Fecal Short-Chain Fatty Acids (SCFAs)

Gas chromatography (GC-2010, Shimadzu, Kyoto, Japan) was used to detect the content of SCFAs [42]. Briefly, 0.1 g of fecal samples were suspended in 0.5 mL of dilute sulfuric acid (H_2_O: 50% H_2_SO_4_ = 4:1, *v/v*). After homogenization (5 min) and centrifugation (13,000× *g*, 10 min), the supernatant was extracted with ethyl ether of equal volume for analysis. Chromatographic analysis conditions were consistent with those described previously [42].

### 2.8. Detection of Immune Cells in Tumor and Spleen by FACS 

The surface marker expression in tumors was analyzed according to the literature [43]. In brief, the tumor and intestinal lamina propria were minced and digested in HBSS with Collagenase IV (Yeasen Biotech Co., Ltd., Shanghai, China) at 37 ℃ for 1 h. A 70 μm cell strainer (BD Biosciences) was used to obtain the single cells through filtering digested tissues. For immune cells from the spleen and mesenteric lymph nodes (MLNs), the spleen was placed directly in a 70 μm cell strainer, and then spleen tissues were gently ground to obtain the single cells using the plunger of a sterile syringe. The spleen cells were added to erythrocyte lysate on ice for 10 min. Single cells were stained for 30 min in the dark at 4 °C with optimized concentrations of anti-mouse antibodies for CD4 (30-F11), CD8 (53-6.7), CD45 (GK1.5), CD25 (PC61.5), CD19 (1D3), and NK1.1 (PK136). For intracellular staining, cells were fixed and permeabilized with a Foxp3/Transcription Factor Staining Buffer Set (eBioscience) after surface staining, and then stained for 30 min in the dark at 4 °C with anti-mouse antibodies including Foxp3 (150D), IL-17 (eBio17B7), and IFNγ (XMG1.2). After washing twice, cells were resuspended in the FACS buffer and measured using a FACS Calibur flow cytometer (BD Franklin Lakes, NJ, USA). The data were analyzed with the FlowJo software (Version 10.6.2, TreeStar, Inc., Ashland, OR, USA).

### 2.9. Determination of Serum IgA and LPS 

Immunoglobulin A (IgA) and lipopolysaccharide (LPS) in serum were measured with the ELISA kits (Invitrogen, Carlsbad, CA, USA) according to the manufacturer’ instructions. 

### 2.10. Analysis of Gut Microbiota

We characterized the composition of the bacterial community in fecal samples using 16S rRNA gene amplicon sequencing. After a 1 week treatment with jujube powder, feces of mice were collected, and the QIAamp DNA Stool Mini Kit (Qiagen, Germany) was applied to extract the genomic DNA according to the instructions of the manufacturer. PCR targeting the V3–V4 region of the 16S rRNA gene was executed, and subsequent amplicon sequencing was performed according to the standard protocols by Shanghai Majorbio Bio-Pharm Technology Co., Ltd. (Shanghai, China). as previously described [26]. The sequences of bacteria were uploaded to the NCBI Sequence Read Archive with accession number SUB9454167 (https://www.ncbi.nlm.nih.gov/sra/SUB9454167) and it will be accessible after 1 May 2022.

### 2.11. RNA-Sequencing and Data Analysis

TRIzol^®^ Reagent was applied for extraction of total RNA from the MLNs following the instructions of the manufacturer (Invitrogen). RNA-Seq analysis was performed with the Illumina HiSeq xten/NovaSeq 6000 sequencer (2 × 150 bp read length) by Shanghai Majorbio Bio-Pharm Technology Co., Ltd. The reads were then processed and analyzed according to [44,45,46]. To identify the differences in gene expression, the transcripts per million reads (TPM) method was used to calculate the expression level of each transcript. Moreover, functional enrichment analysis was performed to identify significant differential genes in GO terms at a *p*-value ≤ 0.05

### 2.12. Bioinformatics Analysis

The alpha diversity was analyzed using the online Majorbio I-Sanger Cloud Platform (www.i-sanger.com). The Euclidean distance was calculated using R package ade4 for beta-diversity analysis, and the key OTUs (operational taxonomic units) which influence PCA clustering were extracted. BugBase was applied to predict the microbiome phenotypes. A co-occurrence network based on the spearman correlation matrix was constructed using the “WGCNA” R package [47]. OTUs with relative abundance >0.1% were used here. The minimum module size was set to 20, while the merge cut height was set to 0.25. The soft threshold was used to ensure a scale-free network. Pearson’s coefficient correlations were calculated to assess the relationships between the OTUs and the production of SCFAs. Permutation analysis of variance (PERMANOVA) using the adonis function in the vegan package with default settings was applied to evaluate community dissimilarity. Pairwise differences were calculated using the ‘pairwise.perm.manova’ function from the package RVAideMemoire.

### 2.13. Statistics

We performed all statistical analysis using GraphPad Prism 8.0 (GraphPad Softioare Inc., San Diego, CA, USA). The two-tailed Student’s *t*-test was used when comparing two groups, and one-way ANOVA was used when making multiple comparisons. All graphs show mean and error bars representing SEM; a *p*-value < 0.05 was considered to be statistically significant, while a *p*-value < 0.01 indicated high statistical significance (* *p* < 0.05, ** *p* < 0.01, *** *p* < 0.001, ns: not significant).

## 3. Results

### 3.1. Surface Properties of Jujube Powders

**Figure 1:**
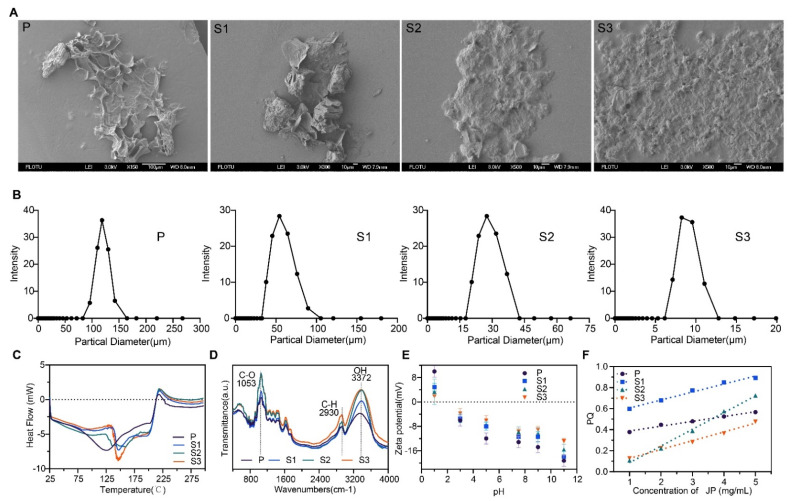
Morphological and surface properties of jujube powder characterized by (**A**) SEM micrographs, (**B**) particle size distributions, (**C**) DSC, (**D**) FTIR spectra, (**E**) zeta potential, and (**F**) hydrophobicity.

Figure 1A shows the SEM images of the jujube powders of different particle sizes. A complete surface was retained for sample P, indicating the existence of original structures. Compared with sample P, major changes can be identified from the surface of ultrafine powders S1, S2, and S3, i.e., the lamellar structure at the surface almost disappeared and the original tissue structure was destroyed. When the particle size was below 10 μm, the internal structure was exposed, which enhanced interparticle attraction and, thus, particle agglomeration.

Figure 1B shows the size distribution of the jujube powders. Sample P exhibited the broadest Z-average size distribution with one peak occurring at 80–160 μm, whereas S1, S2, and S3 had average distributions of 40–110 μm, 15–45 μm, and 6–13 μm, respectively. Accordingly, the Z-average sizes of samples P, S1, S2, and S3 were 137 ± 39 μm, 65 ± 28, 36 ± 4, and 9 ± 0.6 (μm), respectively. Figure 1C shows that all samples exhibited a similar DSC spectrum characterized by a distinct endothermic peak and one exothermic peak. Samples S1, S2, and S3 had a distinct heat absorption peak at 150 °C, whereas the peak of the P sample was broadened and moved forward to 125 °C. This may be attributed to the endothermic effect caused by protein decomposition. One small exothermic peak at 225 °C of all samples may be attributed to ash decomposition. Figure 1D shows that the FTIR spectra of all samples had an absorption peak at 3372 cm^−1^, which is the stretching vibration peak of O–H in natural polysaccharides and phenols. The absorption peak near 290 cm^−1^ refers to the C–H stretching vibration on –CH2 or –CH3 in polysaccharide compounds [38,48,49]. The absorption peak at 1053 cm^−1^ indicates the stretching vibration of C–O from the carbohydrates, such as polysaccharides [50]. As shown in Figure 1E,F, the increase in the jujube powder particle size magnified the zeta potential but reduced the hydrophobicity. Table S1 presents the specific surface area, aqueous solubility, and water holding capacity (WHC) of the ultrafine powders in comparison with those corresponding to coarse powders. The results show apparent changes in the morphology and properties of jujube powder with different particle size.

### 3.2. Changes in Gut Microbiota of Mice with Jujube Powder

**Figure 2:**
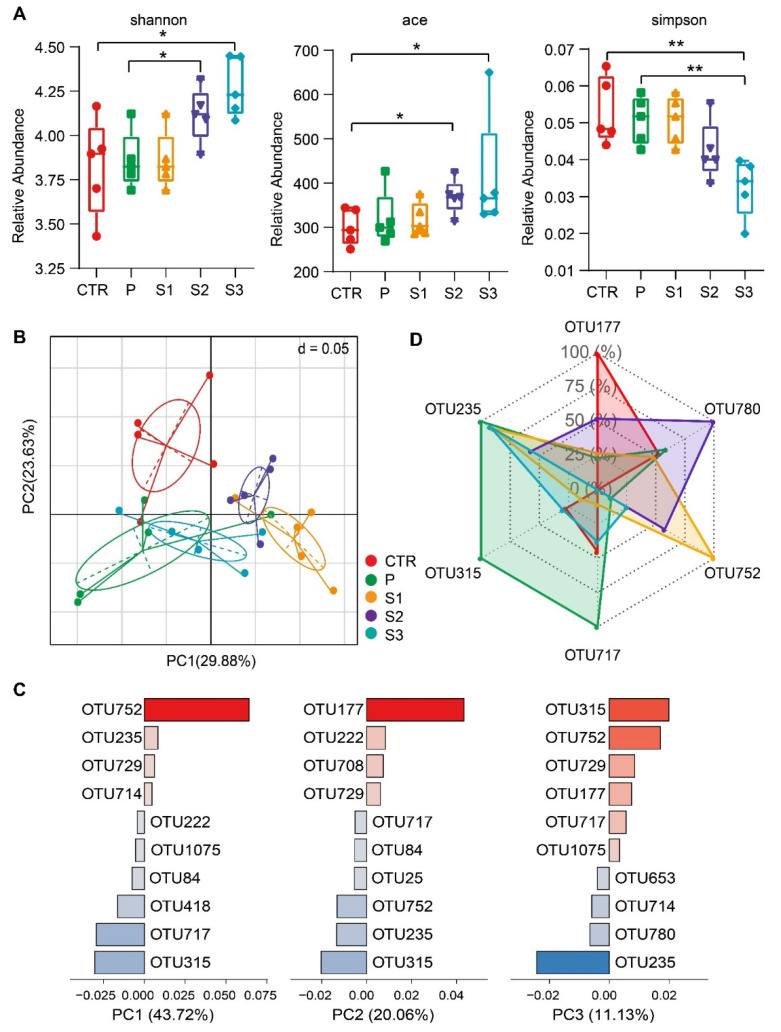
Alterations of the α-diversity and β-diversity in microflora of mice after oral supplement with JP. (**A**) Microbial alpha diversity shown in different indices: Shannon, Ace, and Simpson indices. (**B**) β-diversity analysis of microflora using principal component analysis (PCA) based on OTUs. (**C**) The contribution of key OTUs with the greatest impact on PCA clustering. (**D**) Radar chart of the key OTUs in different groups. * *p* < 0.05, ** *p* < 0.01.

The gut microbiota communities of mice were characterized after 1 week administration of JP by 16S rRNA gene sequencing covering the V3–V4 regions. As shown in Figure 2A, administration of S2 and S3 significantly increased the alpha-diversity index in terms of both microbial richness (Ace) and diversity (Shannon), as compared to the control group (CTR). The Simpson index also reflects the microbial richness. This index is the probability that two individuals chosen randomly from the community belong to identical species [51]. Therefore, a smaller Simpson index indicates greater microbial richness. The Simpson index decreased significantly in S2 and S3 treatment groups, which is consistent with the previous results. Beta diversity (Figure 2B), as interpreted from principal component analysis (PCA), revealed five separate clusters representing differential microbiotas. Permutational multivariate analysis of variance on Bray–Curtis dissimilarities confirmed the marked changes in the composition of microbiota (*R*^2^ = 0.313, *p* < 0.001). Pairwise tests revealed significant changes in S1, S2, and S3 groups (*p* < 0.05), but not the P group when compared with the CTR group. Furthermore, it was found that six key OTUs (OTU752, OTU717, OTU235, OTU315, OTU780, and OTU177) significantly affected PCA clustering (Figure 2C). As can be seen from Figure 2D, these six core OTUs belong to two family levels: Muribaculaceae (OTU752, OTU717, OTU315, and OTU177) and Lachnospiraceae (OTU235 and OTU780). These results indicate that 1 week oral administration of jujube powder with different particle sizes can significantly change the diversity and structure of intestinal microorganisms in mice.

### 3.3. Taxonomic Analysis of the Gut Microbiota

**Figure 3:**
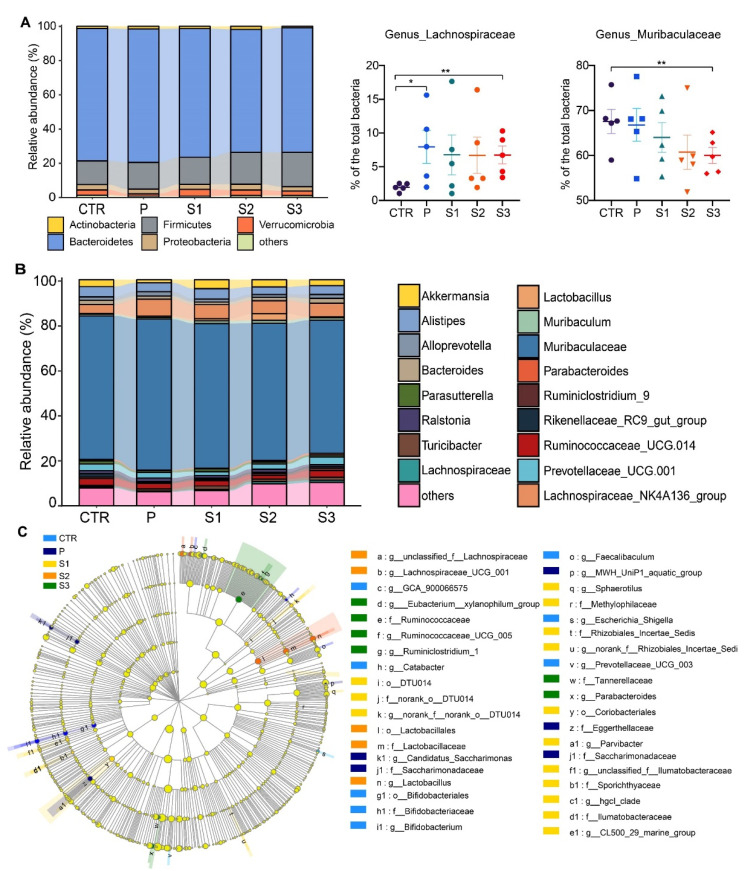
Taxonomic composition at phylum (**A**) and genus (**B**) levels and the difference in dominant microbiotas (**C**). * *p* < 0.05, ** *p* < 0.01.

We further analyzed the taxonomic composition at different levels of intestinal microorganisms. The ratio of Firmicutes to Bacteroidetes (F/B) increased as the particle size fell at the phylum level (Figure 3A). Consistently, microbiotas were differentially abundant in certain genera. All mice fed with JP had more Lachnospiraceae and *Muribaculum* but less Muribaculaceae and Rikenellaceae in the gut microbiota (Figure 3B). Moreover, these changes became more significant in the case of small JP particles. Figure 3C shows the specific differences between groups according to linear discriminant analysis (LDA) and effect size (LEfSe) analysis (LDA score is shown in Figure S3). The results agree well with those shown in Figure 3A,B, i.e., we observed a higher abundance of Lachnospiraceae, Lactobacillaceae, and Ruminococcaceae in mice after oral supplementation with S2 and S3 than in the CTR group. These results indicate that the reduction in particle size rendered jujube powder more effective to increase the population of beneficial microorganisms.

### 3.4. Effects of Jujube Powder (JP) Particle Size on SCFA Production and Gut Microbiota

**Figure 4:**
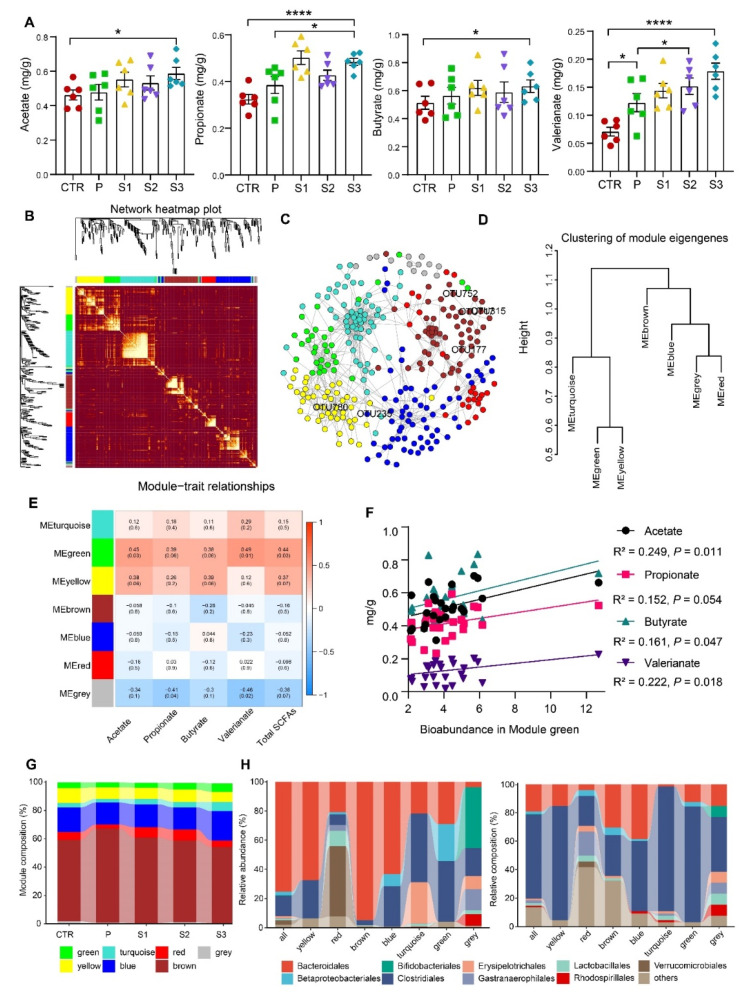
The relationship between the production of SCFAs and OTU clusters. (**A**) SCFA contents in cecum; (**B**) heatmap of topological overlap in the OTU network, where each row and column corresponds to an OTU. The dark red color represents low topological overlap, while the lighter orange color represents higher topological overlap; (**C**) network diagram with nodes colored according to each of the seven microbial clusters; (**D**) hierarchical clustering dendrogram of module eigen-OTUs; (**E**) correlation among modules and traits; (**F**) the regressed relationships between the keystone microbial cluster (green) and production of SCFAs; (**G**) module changes among groups; (**H**) OTU abundance (left panel) and OTU composition (right panel) in each module (*n* = 6). * *p* < 0.05, **** *p* < 0.0001.

Jujube is rich in polysaccharides, dietary fiber, and other complex carbohydrates that can be fermented by microbes to produce SCFAs and provide energy and nutrients to intestinal epithelial cells. SCFAs have many health benefits including regulation of energy metabolism and immune system. Here, we first determined the production of SCFAs after oral supplementation with JP. Figure 4A shows that the administration of JP to healthy mice greatly increased the production of acetate, propionate, butyrate, and valerianate. Next, weighted gene co-expression network analysis (WGCNA) was performed to recognize clusters of OTUs. These clusters are highly correlated with each other. A high-confidence scale-free network was constructed with soft threshold β = 5 and scale-free *R*^2^ = 0.85. Using the Dynamic Tree Cut algorithm, we obtained a total of seven OTU co-occurrence modules, as shown in Figure 4B. Figure 4C shows the relationship among OTUs in different modules and the classification of the five key OTUs we previously identified. Cluster analysis shows that the modules were divided into two categories, among which yellow, green, and turquoise modules belonged to one category, and the other three belonged to the other category (Figure 4D). Compared to other modules, the green, yellow, and brown modules were highly correlated with the production of SCFAs, as shown in Figure 4E. The green and yellow modules were positively correlated with the production of SCFAs, while the trend for the brown module was the opposite. Specifically, the abundance of OTUs in green modules was connected to the production of all SCFAs except propionate (Figure 4F). Figure 4G shows the module compositions in different groups, in which the abundance of green, yellow, and turquoise modules was significantly enhanced in the groups treated with JP and, more importantly, this tendency became more significant as the particle size of JP decreased. Figure 4H shows that these modules were enriched with Clostridiales, consistent with the results obtained from cases S2 and S3, where the abundance of Lachnospiraceae and Ruminococcaceae increased. The above results prove that the changes in gut microbiota were consistent with the production of short-chain fatty acids.

### 3.5. The Effects of JP Particle Size on the Enhancement of αPD-L1 Efficiency against Murine Colon Adenocarcinoma

**Figure 5:**
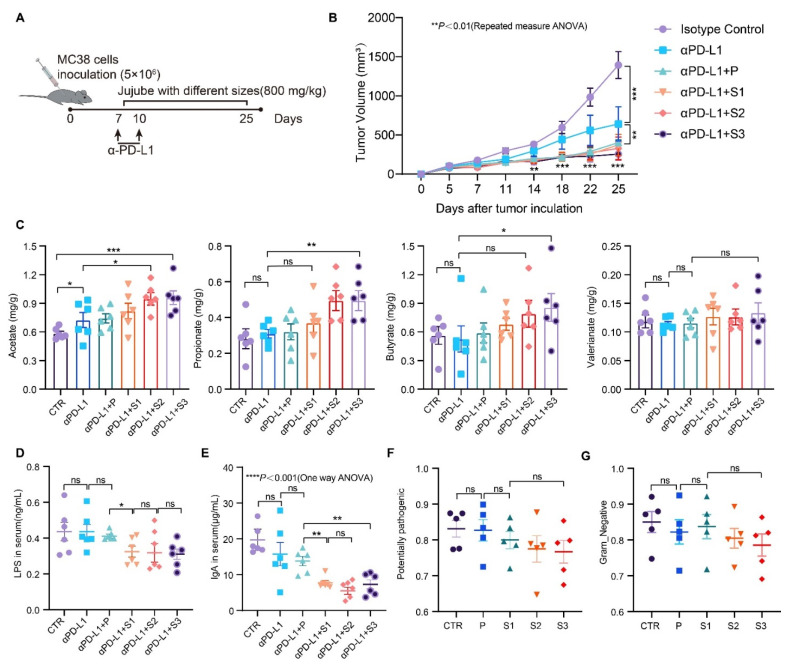
A reduction in JP particle size enhances its efficiency for anti-PD-L1 therapy. (**A**) Animal experiment design; (**B**) growth curve of tumor cells; (**C**) SCFA contents in cecum; (**D**) concentration of LPS in serum; (**E**) concentration of IgA in serum; (**F**) BugBase predicted potentially pathogenic and Gram-negative bacteria (**G**) in the microbial communities (*n* = 6). Data indicate the mean ± SEM; * *p* < 0.05, ** *p* < 0.01, *** *p* < 0.001.

As shown in Figure 5A, in all JP and αPD-L1 combined treatment groups, the tumor growth rate slowed significantly compared to the αPD-L1 group (Figure 5B). Notably, the strongest inhibition of tumor growth took place in the S3 and αPD-L1 joint treatment group (tumor volume data and pictures of tumor-bearing mice are shown in Table S3 and Figure S1). The body weight of the mice increased steadily throughout the experimental period in all groups (Figure S2), indicating that the administration dosages of JP and αPD-L1 were safe. Next, we measured the SCFA level in the cecum on the 25th day. As shown in Figure 5C, the JP with smaller particles resulted in a higher SCFA productivity. To examine the microbe-induced immunologic changes, we measured the LPS (lipopolysaccharide) content in serum with ELISA. As shown in Figure 5D,E, smaller JP particles reduced the serum LPS and immunoglobulin A (IgA) levels. In the BugBase analysis, both Gram-negative bacteria (sources of LPS) (Figure 5G) and potentially pathogenic bacteria (Figure 5F) decreased more significantly in the case of ultrafine JP particles, as compared to the αPD-L1 group. These results indicate that JP may impact the mouse immune response through the metabolites of the gut microbiota such as SCFAs rather than the cellular components of bacteria. The implication of these results is that reducing the particle size of jujube powder is beneficial to enhance αPD-L1 efficiency against murine colon adenocarcinoma.

### 3.6. Analysis of Tumor Immune Infiltration and System Immunity

**Figure 6:**
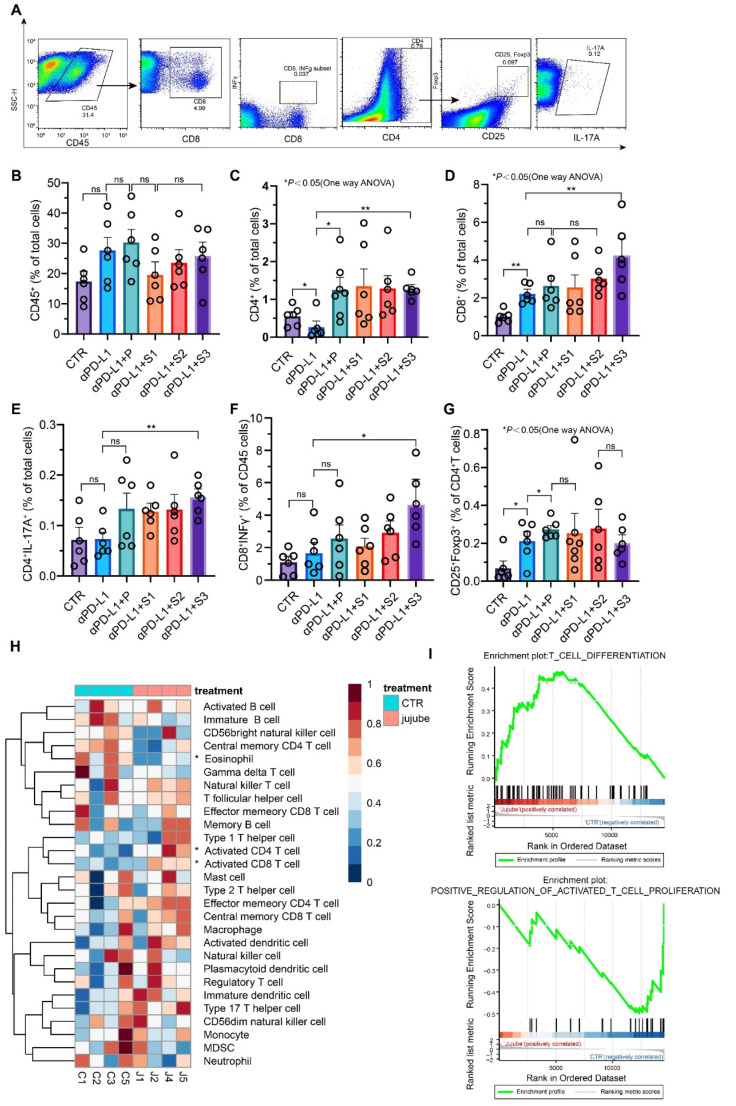
JP enhances the infiltration of immune cells in the TME. (**A**) Representative flow cytometry plots showing the expression of CD45, CD8^+^, Th17, CD4^+^, regulatory T cells (Tregs) and cytotoxic CD8^+^ T cells in tumor tissues; (**B**–**G**) percentage of CD45^+^ cells, CD4^+^ T cells, CD8^+^ T cells, Th17 cells, and cytotoxic CD8^+^ T cells among CD8^+^ T cells and Tregs among CD4^+^T cells in the tumor tissues; (**H**) RNA-seq analysis of mesenteric lymph nodes from wild-type and JP-treated mice (*n* = 4 per group); (**I**) GSEA analysis of T-cell differentiation and activation. The diagram plots the GSEA for two gene sets upregulated in JP-treated groups (left side, Jujube; right side, CTR). The vertical axis in the upper graph denotes the enrichment score (ES), while the barcode plot denotes the position of genes. NES, normalized ES; FDR, false discovery rate. Data indicate the mean ± SEM; * *p* < 0.05, ** *p* < 0.01.

The effects of JP on the tumor-associated immune infiltrates were analyzed by FACS using the gating strategy defined in Figure 6A. Compared with the αPD-L1 group, there was no significant change in total leukocyte abundance in the gavage group (Figure 6B), but the differentiation of T-cell subtypes changed significantly (Figure 6C–G). A higher density of CD8^+^ T cells was observed in S2 and S3 groups (Figure 6D). It is noteworthy that αPD-L1 treatment resulted in a noticeable reduction in CD4^+^ T cells, whereas the administration of JP recovered and improved the ratio of CD4^+^ T cells; moreover, it led to a remarkable increase in Th17 cells, particularly in the S3 group. To understand how SCFAs increase CD8^+^ T cells in tumor-infiltrating lymphocytes, JP-treated and untreated mice mesenteric lymph nodes were isolated for RNA-sequencing (RNA-Seq). In the JP treatment group, many T-cell-associated genes were upregulated, especially the activated CD4^+^ T cells and CD8^+^ T cells, suggesting that JP could promote CD8^+^ T-cell activation in mesenteric lymph nodes (Figure 6H). Gene set enrichment analysis (GSEA) shows that both gene sets directly associated with T cells were significantly upregulated, including T-cell differentiation and T-cell activation in mesenteric lymph nodes after JP treatment (Figure 6I). This also explains the origin of CD8^+^ T cells in tumors. These data suggest that, consistent with the growth rate of tumor volume, JP with smaller particle size can increase the population of CD8^+^ T cells in tumor-infiltrating lymphocytes.

**Figure 7:**
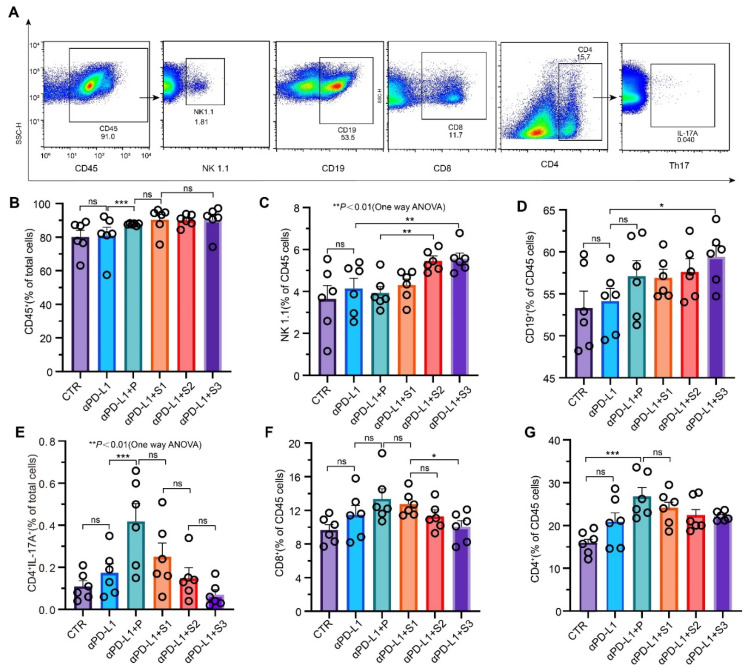
After administration with JP, the gut microbiota shows enhanced systemic immunity. (**A**) Representative flow cytometry plots showing the expressions of CD45, NK 1.1, CD19, Th17, CD4^+^, and CD8^+^ T cells in spleen tissues; (**B**–**G**) percentages of CD45 cells, NK 1.1 cells, CD19 cells, Th17 cells, CD8^+^ T cells, and CD4^+^Tcells in spleen tissues. Data indicate the mean ± SEM; * *p* < 0.05, ** *p* < 0.01, *** *p* < 0.001.

A systematic antitumor immune response, in terms of the composition of immune cells in spleen, was determined by FACS. As shown in Figure 7A–D, the percentage of total leukocytes (CD45), NK cells (NK 1.1), and B cells (CD19) increased as the particle size of JP was reduced. Given the fact that NK cells destroy foreign cells (e.g., tumor cells) without prior sensitization, it is concluded that the systematic immunity was enhanced by the uptake of JP. Interestingly, the percentage of CD8^+^ T cells, CD4^+^ T cells, and Th17 cells did not show a significant change in spleen. In fact, the levels fell slightly with a decrease in particle size (Figure 7E–G), suggesting that the ultrafine JP particles were more advantageous in avoiding excess immune response.

### 3.7. Effects of SCFAs on Immune Cell Composition In Vivo and In Vitro

**Figure 8:**
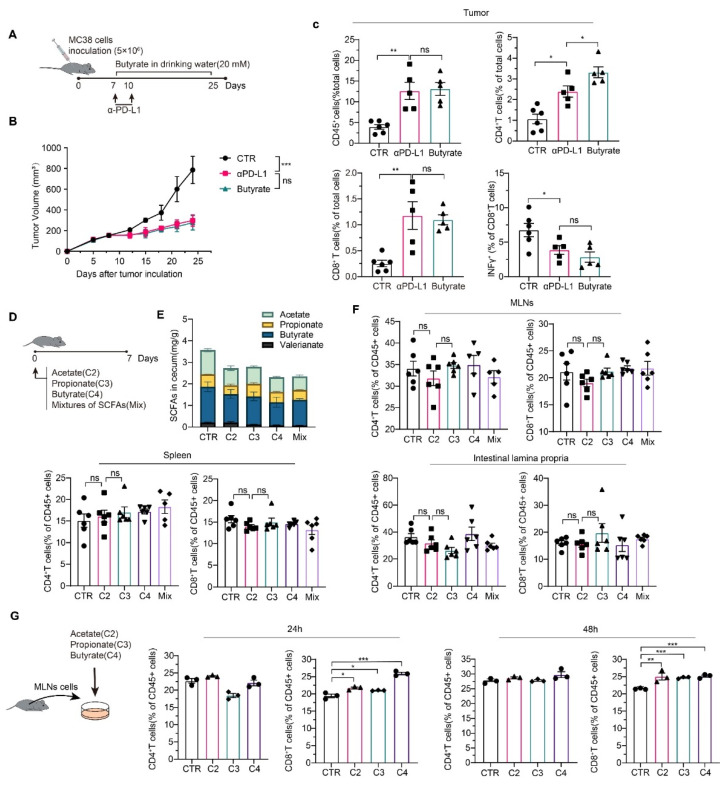
Effects of SCFAs on immune cell composition in vivo and in vitro. (**A**) Animal experiment design (*n* = 6); (**B**) growth curve of tumor cells; (**C**) analysis of tumor-infiltrating lymphocytes by FACS; (**D**) animal experiment design: C57BL/6 mice were treated with 80 mM acetate (C2), propionate (C3), butyrate (C4), and mixtures of SCFAs (40 mM C2, 40 mM C3, and 40 mM C4) in drinking water for 1 week; (**E**) SCFA contents in cecum; (**F**) CD4^+^ T cells and CD8^+^ T cells in MLNs, spleen, and intestinal lamina propria were examined. (**G**) SCFAs directly affect CD4^+^ T cells and CD8^+^ T cells in MLNs in vitro. Data indicate the mean ± SEM; * *p* < 0.05, ** *p* < 0.01, *** *p* < 0.001.

Since it has been reported that butyrate can induce cancer cell apoptosis [52], we first added butyrate to the drinking water of mice during αPD-L1 treatment (Figure 8A). Unexpectedly, oral supplement with butyrate did not enhance αPD-L1 efficiency, as shown in Figure 8B. Analysis of tumor-infiltrating lymphocytes also confirmed the previous results, as there was no significant difference in the ratio of total leukocytes and CD8^+^ T cells between the αPD-L1 group and butyrate + αPD-L1 group (Figure 8C). We also examined the effects of several other SCFAs on the immune system, including the immune cell composition in mesenteric lymph nodes (MLNs), spleen, and intestinal lamina propria (Figure 8D). However, oral supplementation with SCFAs did not increase their content in the cecum (Figure 8E), which also explains why the CD8^+^ T-cell and CD4^+^ T-cell efficiency did not change in the MLNs, spleen, or intestinal lamina propria (Figure 8F). Lymphocytes were isolated from mouse MLNs and cultured with SCFAs in vitro (Figure 8G). Butyrate treatment resulted in an increased proportion of CD8^+^ T cells after incubation of 24 h, while other SCFAs could also directly facilitate the CD8^+^ T cells after a longer incubation (48 h). The effects of SCFAs on CD4^+^ T cells may take a longer incubation time or require higher concentrations. These results indicate that direct supplementation of short-chain fatty acids could not increase their levels in the colon. Therefore, it could not stimulate the differentiation of immune cells in the mesenteric lymph nodes.

## 4. Discussion

Mei Kong and coworkers have shown that glutamine supplementation can block melanoma tumor growth through suppressing epigenetically activated oncogenic pathways, which can serve as a potential dietary intervention [53]. Xiaohuan Guo and coworkers reported that butyrate can directly enhance the CD8^+^ T cell immune response and enhance chemotherapy efficacy [54]. Whether dietary interventions are useful in colorectal cancer remains to be explored. In this study, we discovered that oral administration of jujube powder (JP) is able to ameliorate the mouse gut biota with an enriched population of Lachnospiraceae and Ruminococcaceae, an enhanced production of SCFAs, and an improved tumor immune infiltration and systemic immunity, which collectively contribute to the higher antitumor efficiency of αPD-L1 in vivo. The therapeutic effects were most significant in the case of using ultrafine JP particles. 

Here, we found that the smallest JP particles showed a smoothened spherical-like shape (Figure 1A), which is helpful for its role as a prebiotic [36,55,56]. FTIR results showed that ultrafine grinding did not change the group structure of jujube powders or the main components. However, compared with the ordinary powder, the absorption peak strength of ultrafine jujube powder was intensified, which indicates that the number of functional groups increased. This phenomenon could be due to an increased specific surface area [57]. It is also noteworthy that the magnitude of zeta potential was reduced as the particle size decreased, indicating an enhanced uptake of JP particles by the gut microbiota. These microbes adhere to the particle surface and release hydrolytic enzymes to hydrolyze macromolecular nutrients before absorption. Therefore, changes in the surface chemistry of substrates may affect the adhesion behavior of microbes. Min and Akbulut demonstrated that the surface chemistry of substrate could influence the kinetics and thermodynamics of bacterial adhesion [58,59]. Cooper et al. showed that the bacterial adhesion was higher on hydrophobic surfaces [60,61]. The adhesive forces between substrate and bacterium arise through van der Waals and electrostatic double-layer interactions [62]. On the basis of the above discussion, we conjecture that ultrafine jujube powder is beneficial for microbial adhesion and nutrient utilization.

In particular, we observed that the abundance of Lachnospiraceae and Ruminococcaceae increased in groups treated with ultrafine powders. In order to exclude the influence of microbes carried by jujube powder itself on the results, we detected the microbial purity of JP (Table S4). The total amount of microbes in JP was below 10 CFU/g, which is much less than that reported in the gut, being 4 × 10^13^ [63,64]. Moreover, the *Clostridium* genus were not found in JP. We, thus, concluded that the changes in gut microbiota were contributed by the JP rather than the microbes on the surface of JP. These two species are thought to be butyrogenic populations belonging to clostridial cluster XIV (Lachnospiraceae) and cluster IV (Ruminococcaceae) [65], which lead to an improvement of nondigestible carbohydrate fermentation profiles and an increase in SCFA production. This flora composition may, thus, contribute to increased energy harvest from the diet [66]. Hence, we examined the content of SCFAs in the cecum. Consistently, the reduction in particle size accelerated SCFA production, which was consistent with the changes in bacterial flora composition. WGCNA further pointed out a relationship between Clostridiales and SCFA production, supporting the previous assertion. Higher stool SCFA concentrations in adults have been linked to greater intake of dietary fiber, particularly from foods rich in fermentable fibers [67]. A large number of studies have shown that the physical form and structure of dietary fiber (DF) greatly influence its microbiota-modulating activity [56,68]. Moreover, our study showed that particle size had an impact on the availability of DF in jujube powder (Table S2), which in turn may influence the fermentability of DF [69], boosting SCFA production [70]. 

The differential structure of the gut microbiota may influence therapeutic responses to ICI through impacting the tumor microenvironment, including infiltrating immune cells that can stimulate (such as CD8^+^ T cells) an immune response. We observed that the responders to anti-PD-1 therapy are rich in Clostridiales, Ruminococcaceae, and Lachnospiraceae [4,6,35], which are positively correlated with CD8^+^ T-cell infiltration in the tumor [6]. Recently, Tomita et al. showed that *Clostridium butyricum*, specialized in producing butyrate, may have a positive impact on the therapeutic efficacy of ICI in patients with lung cancer [71]. Here, we observed an increase in these species in the S3 group. Higher levels of SCFAs produced by these microbes further stimulate immune cell differentiation in the gut. After being primed by dendritic cells (DCs) in draining mesenteric lymph nodes (MLNs) of the gut, B and T cells, including Tregs and Th17 cells, could circulate systemically to facilitate immune responses at distant sites [72]. A significant positive correlation has been confirmed between the CD8^+^ T cell infiltrate in the tumor and abundance of the Clostridiales and Ruminococcaceae [6]. Given that Th1 cells (CD4^+^ IFNγ^+^) play a key role in activating CD8^+^ T cells, we also analyzed the tumor-infiltrating CD4^+^ T cells. FACS results revealed a significant increase in the infiltration of CD4^+^ T cells in mice treated with JP. Administration of the combination treatment with anti-PD-L1 antibody and JP increased the population and antitumor function of DCs, thereby promoting recruitment of activated CD8^+^ T cells to the tumor microenvironment. In addition, we also found that direct supplementation of SCFAs did not improve tumor-infiltrating lymphocytes, which may be due to digestion and absorption after oral administration, preventing an effect in the intestine. SCFAs have also been shown to influence immunity via IgA production by plasma cells, which may affect local immunity [73]. The reduction in plasma IgA secretion after JP administration suggests a more complete intestinal mucosal barrier.

Although our current research achieved encouraging results in syngeneic tumor models, complex genetically engineered mouse models will be valuable for predictive evaluation of tumor immunotherapies in the preclinical setting, which can accurately reflect the autochthonous tumor microenvironment. Different mouse models, as discussed by Olson [74], will be investigated in our future work.

## 5. Conclusions

Given all the findings of this work, we conclude that the administration of ultrafine jujube powder (JP) alters intestinal flora composition as evidenced by the high abundance of Clostridiales, including Ruminococcaceae and Lachnospiraceae, as well as enhanced SCFA production. Ultrafine JP particles greatly improved the response of anti-PD-L1 treatment, as shown by an intensified infiltration of CD8^+^ T cells into the TME and maintenance of the intestinal mucosal barrier. Our study indicates that, combined with nutritional interventions during the treatment of immune checkpoint inhibitors, the effectiveness of tumor immunotherapy can be improved. Future efforts will be directed at understanding the molecular details of the interaction among nutrients, gut microbiota, and immune response. Nutritional intervention in cancer prevention is also worthy of attention.

## Data Availability

The 16S rDNA sequencing dataset was deposited in the SRA database of NCBI with accession ID SUB9454167. Other data that support the findings of this study are available upon reasonable request from the corresponding author.

## Supplementary Materials

The following are available online at https://www.mdpi.com/article/10.3390/cancers13163987/s1, Table S1: Physicochemical properties of P, S1, S2 and S3, Table S2: Effects of ultrafine grinding on the contents of dietary fiber in JP, Table S3: Tumor volume (mm3), Table S4: jujube powder’s microbial purity, Figure S1: Picture of tumor-bearing mice among different groups, Figure S2: Body weight were measured twice weekly (*n* = 6), Figure S3: LDA Score of LEfSe analysis.
