# Supplementary material for: Ultrafine Jujube Powder Enhances the Infiltration of Immune Cells during Anti-PD-L1 Treatment against Murine Colon Adenocarcinoma
Source: Cancers (Basel). 2021 Aug 7;13(16):3987. doi: 10.3390/cancers13163987 (PMC8394940; doi:10.3390/cancers13163987)
Supplement: Supplementary file 1 [file cancers-13-03987-s001.zip › cancers-1264168-supplementary.pdf]

Supplementary Materials:

Table S1. Physicochemical properties of P, S1, S2 and S3

Samples	Angle of repose /°	Bulk density /g·cm^{-3}	Water holding capacity (%)	Swelling ratio(mL/g)	Solubility (%)	Moisture absorption (%)	Surface area (m^2/g)
P	20.70 ± 0.82	0.58 ± 0.06	104.39 ± 1.56	4 ± 0.18	40.23 ± 1.23	28.54 ± 1.01	0.98 ±0.02
S1	37.48 ± 0.23	0.55 ± 0.03	97.15 ± 1.89	4.5 ± 0.23	48.52 ± 1.87	30.87 ± 0.98	1.39 ±0.03
S2	46.08 ± 0.67	0.47 ± 0.02	85.44 ± 1.19	5.25 ± 0.76	52.31 ± 0.99	33.48 ± 0.86	2.11 ±0.09
S3	48.81 ± 0.85	0.47 ± 0.01	84.13 ± 0.84	5.5 ± 0.54	59.37 ± 1.46	35.93 ± 0.24	3.35 ±0.06

Table S2. Effects of ultrafine grinding on the contents of dietary fiber in JP

Samples	TDF (%)	IDF (%)	SDF (%)	IDF/SDF
P	20.50 ± 0.98	17.83 ±0.15	2.68 ± 0.06	6.66 ± 0.54
S1	20.46 ± 0.78	17.42 ± 0.28	3.04 ± 0.12	5.72 ± 0.18
S2	20.08 ± 0.16	15.35 ± 0.25	4.73 ±0.08	3.24 ± 0.64
S3	20.97 ± 0.83	15.91 ± 0.68	5.06 ±0.34	3.14 ± 0.46

Note: TDF, total dietary fiber; IDF, insoluble dietary fiber; SDF, soluble dietary fiber.

Table S3. Tumor volume (mm^3)

Groups	Day 5	Day 7	Day 11	Day 14	Day 18	Day 22	Day 25
Control	101.62 ± 28.25	173.94 ± 10.76	296.74 ± 37.71	382.24 ±27.43	545.79 ± 78.75	984.05 ± 115.49	1393.79 ± 172.64
αPD-L1	99.99 ± 20.15	168.54 ± 20.22	190.43 ± 41.48	297.58 ± 73.39	441.90 ± 122.85	559.37 ±192.51	641.51 ± 219.46
αPD-L1+P	97.75 ± 20.21	161.13 ± 18.26	176.65 ± 28.12	233.90 ± 33.56	256.62 ± 36.42	355.08 ± 55.50	430.53 ± 74.41
αPD-L1+S1	96.56 ± 11.35	155.21 ± 19.64	160.91 ±29.08	185.09 ± 41.33	221.53 ± 56.76	256.17 ± 81.50	374.15 ± 94.29
αPD-L1+S2	97.12 ± 13.19	154.46 ± 14.95	165.51 ± 22.91	180.91 ± 35.76	229.50 ± 50.51	271.91 ± 85.50	328.73 ± 95.10
αPD-L1+S3	94.69 ± 9.99	155.67 ± 35.05	158.80 ± 15.53	171.06 ± 31.64	215.14 ± 51.61	228.55 ± 73.56	255.89 ± 76.71

Table S4. jujube powder's microbial purity

Test items	Test method	Test results
Total plate count, CFU/g	GB 4789.2-2016	＜10
Escherichia coli, CFU/g	GB 4789.38-2012	＜10
Salmonella, /25g	GB 4789.4-2016	Non-Detected
Staphylococcus aureus, CFU/g	GB 4789.10-2016	＜10
Clostridium, /g	GB/T4789.12-2003 USP 43 <62>	Non-Detected

Microbiological detection of jujube powder: According to the National Food Safety Standard GB 4789.2-2016, we applied plate colony counting medium (PCA) to determine the contents of total plate count in jujube powders. The most probable number (MPN) counting method was applied to determine the contents of *Escherichia coli* according to GB 4789.3-2012. Biochemical reaction-serological identification was used to determine the contents of *Salmonella* according to GB 4789.4-2016. Baird-Parker PCA method was used to determine the contents of *Staphylococcus aureus* according to GB 4789.10-2016. Biochemical reaction-serological identification was used to determine the contents of *Clostridium* according to GB/T4789.12-2003 and USP 43 <62>. Briefly, 25g samples were added to distilled water for homogenization. The mixture was diluted 10 times and 2-3 appropriate dilutions were picked up for culture. Then, 1 mL of appropriate dilutions were added into the different flat plates and cultured at 25-28 ℃ for 7 days. Finally, colony count was used to indicate microbial purity of jujube powder."

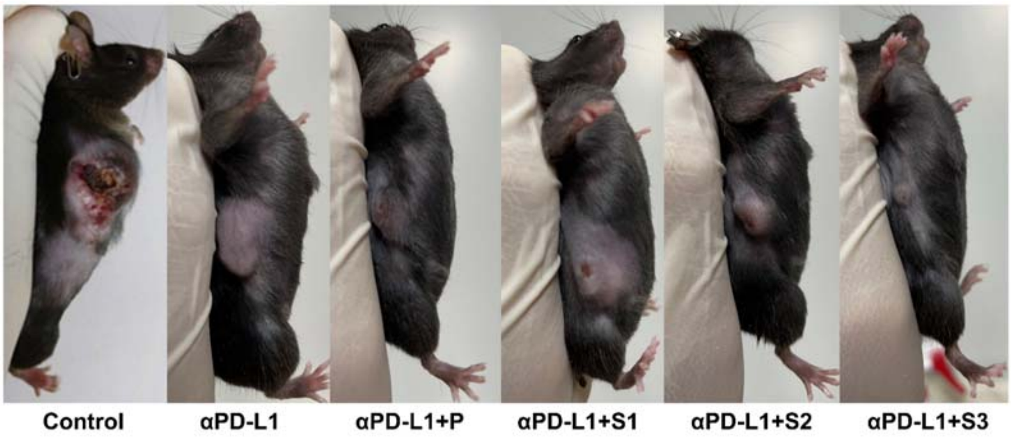

Figure S1. Picture of tumor-bearing mice among different groups.

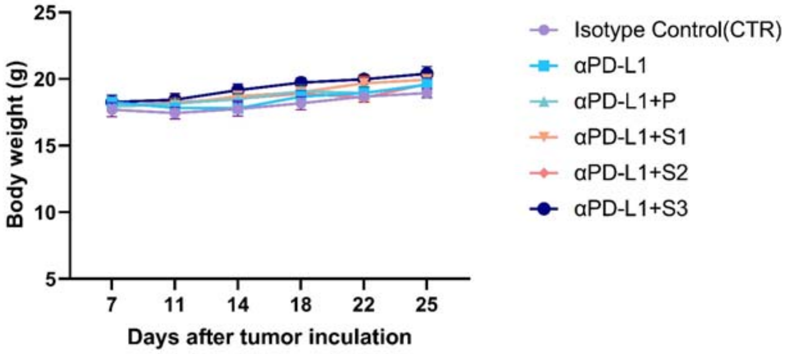

Figure S2. Body weight were measured twice weekly (n = 6).

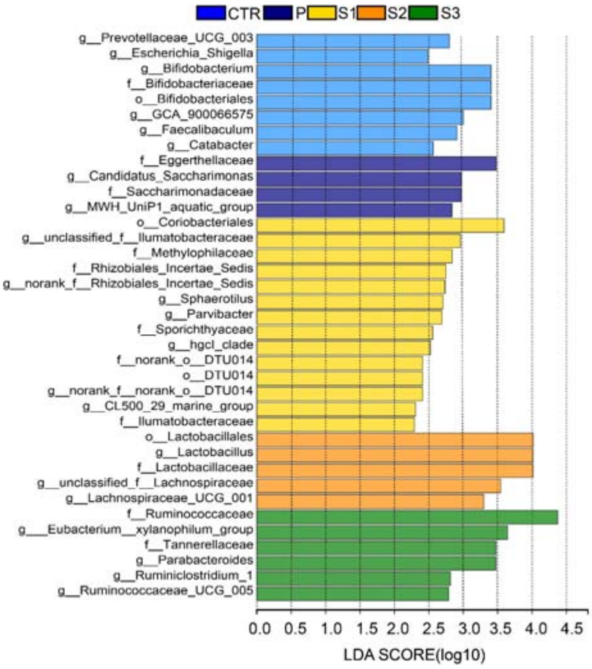

Figure S3. LDA Score of LEfSe analysis.
